# Molecular epidemiology, pathogenicity, and structural analysis of haemoglobin variants in the Yunnan province population of Southwestern China

**DOI:** 10.1038/s41598-019-44793-0

**Published:** 2019-06-04

**Authors:** Jie Zhang, Peng Li, Yang Yang, Yuanlong Yan, Xiaohong Zeng, Dongmei Li, Hong Chen, Jie Su, Baosheng Zhu

**Affiliations:** 1 Genetic Diagnosis Center, Yunnan Provincial Key Laboratory for Birth Defects and Genetic Diseases, The First People’s Hospital of Yunnan Province, Yunnan Province, China; 2 Department of Hematology, The First People’s Hospital of Yunnan Province, Yunnan Province, China; 3 Department of Pediatrics, The Affiliated Hospital of Kunming University of Science and Technology, Kunming, Yunnan China

**Keywords:** Disease genetics, Molecular medicine

## Abstract

Abnormal haemoglobin (Hb) variants result in the most commonly inherited disorders in humans worldwide. In this study, we investigated the molecular epidemiology characteristics of Hb variants, along with associated structural and functional predictions in the Yunnan province population of Southwestern China. A total of 41,933 subjects who sought haemoglobinopathy screening were included. Based on bioinformatics and structural analysis, as well as protein modeling, the pathogenesis and type of Hb genetic mutations were characterized. Among all individuals studied, 328 cases (0.78%) were confirmed as carriers of Hb variants, with 13 cases (0.03%) presenting α-globin variants, 313 (0.75%) β-globin variants, and two δ-globin variants. A total of 19 different mutations were identified, including three novel mutations. In addition, 48 cases of αα^CS^ mutations and 14 cases of Hb H or Hb Bart’s were found. The isoelectric point, evolutionary conservation, and genotype-phenotype correlation for these mutations were predicted. Additionally, secondary and tertiary protein structure modeling were performed for three selected mutations. In conclusion, the prevalence of Hb variants in the Yunnan population is much higher than other regions of China. Complete characterization of these Hb variants is essential for generating a rational strategy to control the haemoglobinopathies in this region.

## Introduction

Haemoglobinopathies are autosomal recessive disorders, popular in former malaria-affected areas including the Mediterranean, the Middle-East, Southern China, and South-East Asia regions^1^. To date, more than one thousand different mutant alleles of haemoglobinopathies have been documented and archived in the Globin Gene Server, Hbvar (http://globin.bx.psu.edu/). Moreover, haemoglobinopathies can be further divided into two groups that can lead to abnormal globin chain synthesis: thalassaemia and Hb variants. Thalassemia is characterized by the reduction or the entire elimination of normal globin chain production. In Southern China, the average frequency of α-thalassemia (OMIM604131) is 6.99% to 45.04% and β-thalassemia (OMIM613985) is 2.70% to 6.66%^2^. Whereas, the Hb variant subtype is characterized by structural protein abnormalities in the globin chains, including α-, β-, δ-, and γ-globins^3^. Most Hb variants have no meaningful clinical significance and are occasionally detected during routine testing, such as pre-pregnancy examinations or during genetic counseling by capillary electrophoresis (CE)^4^.

The Yunnan province is a multi-ethnic region in Southwest China, acting as an important gateway that links Southeastern Asia with Southern China. As of 2017, the average frequency of α-thalassemia in the Yunnan population is 6.99% and β-thalassemia is 2.70%^2^. Additionally, more than 20 types of β-globin mutations have been previously identified in this population^5^. Notably, a high thalassemia carrier frequency of 49.5% was found among the Dai ethnic group in the Southern Yunnan province^6^. Interestingly, among all the high haemoglobinopathy incidence regions of Southern China, the highest frequency of Hb variants was found in the Yunnan population in 1986 with a frequency of 6.06%^7^. Nonetheless, for the past thirty years, only few studies have reported on the frequency and characterization of Hb variants based on larger population samples in Southwest China.

Importantly, the frequency and types of Hb variants differ considerably with geographical location and ethnic group^8^. Thus, the diagnosis of Hb variants is an important basic investigation for haemoglobinopathy screening and birth defect prevention. In our study, we performed a complete molecular epidemiological study of Hb variants for 41,933 consecutive samples from the Yunnan province using CE, molecular, and bioinformatics analyses. Consistently, a more accurate spectrum and frequency of mutations leading to Hb variants in the Yunnan population were determined. Subsequently, the isoelectric point (pI), evolutionary amino acid conservation among variants, and characterization of CE data from mutations underlying these Hb variants, were compared and structurally modeled to further determine the pathogenicity and structural features of these variants.

## Material and Methods

### Study subjects and hematological analysis

A total of 41,933 subjects (9,960 men and 31,973 women, 1 to 45 years of age), who sought Hb variant screening programs (programs conducted in the First Peoples’ Hospital of the Yunnan Province, Maternal and Child Health Hospital of Wenshan, Xishuangbanna, Dehong, Lijiang, Lincang, Qujing, Puer, and Zhaotong) during July 2014 to April 2017 had CE performed using Sebia’s free solution CE instruments (Sebia, France). The protocol and information consent for this study were approved by the medical ethics committee of the First Peoples’ Hospital of Yunnan Province, PRC. Clinical investigations were performed according to the tenets of Declaration of Helsinki and informed written consent was obtained from all participants or legal guardians. In brief, venous blood samples were collected from subjects in tubes containing ethylenediaminetetraacetic acid (EDTA). Internal quality control was then performed by analyzing the samples against control materials provided by the manufacturer (Sebia, France). Individuals showing abnormal Hb bands (except Hb A, Hb A_2_ and Hb F) were considered to be positive carriers of Hb variants.

### DNA analysis

Genomic DNA was extracted from whole blood samples of suspected Hb variant carriers using standard Genomic DNA Extraction Kits (Tianlong Bioscience, China). Hb variants were sequenced and analyzed by the following methods: (1) all positive samples were sequenced for β-globin or α-globin genes according to the panel provided by the manufacturer; (2) Samples with a suspected second Hb A_2_ fraction (low levels of Hb A_2_ associated with a visible abnormal band of less than 2.0%) were sequenced for the δ-globin gene^9^; (3) Gap-PCR was used to determine the three most common Chinese α-globin gene deletion mutations [−α^3.7^ (NC_000016.9:g.223300_227103del), −α^4.2^ (NC_000016.9:g.219817_(223755_224074)del), and–^SEA^ (NC_000016.9:g.215400_234700del) for Hb H disease and Hb Bart’s. The polymerase chain reaction (PCR) reverse dot-blot (RDB) assay was used to detect αα^CS^ (HBA2: c.427 T > C), αα^QS^ (HBA2:c.377 T > C), and αα^WS^ (HBA2:c.369 C > G) as previously reported^10^. The primers used and expected product size for β-globin^5^, α-globin^10^, and δ-globin^11^, are shown in Supplementary Table 1.

### Bioinformatics analysis of Hb variant

All the Hb variants found in this study were analyzed by bioinformatics software. Moreover, pI were determined for Hb variants (i.e., monomer) using Kozlowski’s protein isoelectric point (IP) calculator (http://isoelectric.org/)^12^. The evolutionary conservation of mutated amino acid residues was examined using ConSurf (http://bental.tau.ac.il/new_ConSurfDB/)^8^. The pathogenicity of these candidate mutations were evaluated by the web-based HumDiv-trained Polymorphism Phenotyping v2 (PolyPhen-2) (http://genetics.bwh.harvard.edu/pph2/) and the Sorting Intolerant from Tolerant (SIFT) web server (http://sift.jcvi.org) prediction models^13,14^.

### Molecular modeling

Modelling of secondary and tertiary protein structures of the three selected Hb variants were performed by RaptorX^15^. In addition, the tetramer structure was modeled using the SWISS-MODEL workspace program^16^. The 3D structures of selected mutant proteins were translated from the missense variants and the likely atomic interactions around the mutated residues were estimated by PyMOL using the human haemoglobin protein structure as template (PDB code: 1BZ1; http://www.pymol.org)^17^.

## Results

### Frequency and spectrum of Hb variant

Of the 41,933 blood specimens for analysis of Hb variant, a total of 20,142 subjects selected randomly were registered for native place. Among them, 16,050 subjects came from 16 prefecture-level divisions in different geographical areas throughout Yunnan and 4,092 subjects came from other provinces of China (Supplementary Fig. 1).

Among all individuals studied during this period, 483 (1.15%, 483/41,933) were screened positively by CE. After DNA analysis, 328 cases (0.78%, 328/41,933) were confirmed to be carriers of α-, β-, or δ-globin structural variants. Among these 328 cases, 13 (0.03%; 13/41,933) were α-globin variants and 313 (0.75%; 313/41,933) were β-globin variants, 2 were δ-globin variants. Totally 19 different mutations were identified and summarized in Tables 1 and 2. Sequencing of these variants were shown in Supplementary Fig. 2 and some potentially confounding or novel Hb variants were shown in Supplementary Fig. 3. In addition, 48 cases of αα^CS^ and 14 cases of Hb H or Hb Bart’s were found in this study.

**Table 1:** Molecular and electrophoretic data of Hb variants.

Type	Total (n)	Hb A (%)	Hb A_2_ (%)	Hb variant (%)	Zone	pI
Hb Queens (CD34, Leu > Arg, HBA2:c.104 T > G)	5	80.30 ± 0.30	2.13 ± 0.48	17.00 ± 0.42	Z6	8.39
Hb Q-Thailand (CD74, Asp > His, HBA1:c.223 G > C)	3	71.10 ± 5.20	2.73 ± 1.36	25.37 ± 6.64	Z7	8.39
Hb Daneshgah-Tehran (CD72, His > Arg, HBA2:c.218 A > G)	2	64.05 ± 12.66	1.80 ± 0.42	33.20 ± 12.73	Z6	8.39
Hb Galliera I (CD6, Asp > His, HBA2:c.19 G > C)	1	91.90	2.80	5.30	Z7	8.39
Hb I (CD16, Lys > Glu, HBA2:c.49 A > G)	1	79.80	1.90	18.30	Z15	7.08
Hb Thailand (CD56, Lys > Thr, HBA1:c.170 A > C)	1	75.60	2.00	22.40	Z12	7.72
Hb E (CD26, Glu > Lys, HBB:c.79 G > A)	281	71.72 ± 5.11	3.51 ± 0.33	23.90 ± 2.92	Z4	7.43
Hb New York (CD113, Val > Glu, HBB:c.341 T > A)	15	53.21 ± 4.54	2.89 ± 0.33	42.89 ± 4.62	Z11	6.20
Hb J-Bangkok (CD56, Gly > Asp, HBB:c.170 G > A)	5	46.20 ± 0.45	2.60 ± 0.16	51.04 ± 0.55	Z12	6.20
Hb J-Lome (CD59, Lys > Asn, HBB:c. 180 G > C)	2	46.25 ± 0.49	2.60 ± 0.00	51.15 ± 0.49	Z13	6.20
Hb J-Kaohsiung (CD59, Lys > Thr, HBB:c.179 A > C)	2	45.25 ± 1.63	2.75 ± 0.49	51.20 + 0.00	Z13	6.20
Hb D-Los Angeles (CD121, Glu > Gln, HBB:c.364 G > C)	3	55.60 ± 2.50	3.27 ± 0.12	39.23 ± 1.38	Z6	6.93
Hb G-Copenhagen (CD47, Asp > Asn, HBB:c.142 G > A)	1	55.10	2.90	42.0	Z5	6.93
Hb G-Coushatta (CD22, Glu > Ala, HBB:c.68 A > C)	1	54.30	2.90	42.80	Z6	6.93
Hb Hope (CD136, Gly > Asp, HBB:c.410 G > A)	1	52.10	4.10	42.80	Z10	6.20
Hb Köln (CD98, Val > Met, HBB:c.295 G > A)	1	91.20	3.80	3.70	Z4	6.49
Hb Yunnan (CD49, Ser > Pro, HBB:c.148 T > C)	1	50.40	5.60	44.0	Z8	6.49
Hb A_2_-Puer (CD131, Gln > Glu, HBD:c.394 C > G)	1	97.4	1.30	1.40	Z4	6.87
Hb A_2_-Yunnan (CD65, Lys > Asn, HBD:c.198 G > T)	1	98.0	1.30	0.70	Z6	6.87

Notes: Hb, haemoglobin; pI, isoelectric point. In order to avoid interference, the data were analyzed only for heterozygous. Compound heterozygous or homozygous were not included in the analysis. The pI of normal β-globin, α-globin, and δ-globin were, 6.49, 8.12, and 7.42, respectively.

**Table 2:** The pathogenicity, evolutionary conservation, and structural analysis of Hb variants POLY: PolyPhen-2.

Type	SIFT	POLY	Conservation (score)	Previous description	Structure change^a^
Hb Queens (HBA2:c.104 T > G)	benign	benign	variable (1)	normal (24)^b^	1% α-helix add, 1% coil reduce
Hb Q-Thailand (HBA1:c.223 G > C)	deleterious	damaging	conserved (8)	normal (25)	2% α-helix add, 2% coil reduce
Hb Daneshgah-Tehran (HBA2:c.218 A > G)	deleterious	possibly damaging	average (6)	—	2% α-helix add, 2% coil reduce
Hb Galliera I (HBA2:c.19 G > C)	deleterious	damaging	conserved (8)	—	3% α-helix add, 3% coil reduce
Hb I (HBA2:c.49 A > G)	deleterious	benign	conserved (7)	normal (32)	2% α-helix add, 2% coil reduce
Hb Thailand (HBA1:c.170 A > C)	deleterious	benign	average (4)	normal^c^ (26)	1% α-helix add, 1% coil reduce
Hb E (HBB:c.79 G > A)	deleterious	damaging	average (6)	normal (23)	4% α-helix reduce, 4% coil add
Hb New York (HBB:c.341 T > A)	deleterious	damaging	conserved (7)	normal (29)	2% α-helix reduce, 2% coil add
Hb J-Bangkok (HBB:c.170 G > A)	deleterious	possibly damaging	conserved (7)	normal (27)	3% α-helix reduce, 3% coil add
Hb J-Lome (HBB:c. 180 G > C)	deleterious	damaging	variable (3)	normal (28)	4% α-helix reduce, 4% coil add
Hb J-Kaohsiung (HBB:c.179 A > C)	deleterious	damaging	variable (3)	mild anemia^d^ (30)	2% α-helix reduce, 2% coil add
Hb D-Los Angeles (HBB:c.364 G > C)	benign	benign	variable (2)	mild anemia (33)	3% α-helix reduce, 3% coil add
Hb G-Copenhagen (HBB:c.142 G > A)	benign	benign	conserved (7)	normal (35)	4% α-helix reduce, 4% coil add
Hb G-Coushatta (HBB:c.68 A > C)	benign	benign	variable (1)	normal (36)	3% α-helix reduce, 3% coil add
Hb Hope (HBB:c.410 G > A)	deleterious	possible damaging	variable (3)	normal (37)	2% α-helix reduce, 2% coil add
Hb Köln (HBB:c.295 G > A)	deleterious	damaging	conserved (9)	mild anemia (34)	2% α-helix reduce, 2% coil add
Hb Yunnan (HBB:c.148 T > C)	deleterious	possibly damaging	conserved (7)	—	3% α-helix reduce, 3% coil add
Hb A_2_-Puer (HBD:c.394 C > G)	deleterious	benign	conserved (7)	—	2% α-helix add, 2% coil reduce
Hb A_2_-Yunnan (HBD:c.198 G > T)	deleterious	benign	variable (3)	—	no change

Conservation score: variable (1–3), average (4–6), or conserved (7–9). ^a^The protein secondary structure was predicted by RaptorX. The α-helix, β-sheet, and coil of normal α-globin were 68%, 0%, and 31% respectively. The α-helix, β-sheet, and coil of normal β-globin were 69%, 0%, and 30% respectively. The α-helix, β-sheet, and coil of normal δ-globin were 65%, 0%, and 34% respectively. ^b^reference cited. ^c^Hb Thailand is associated with–^SEA^. ^d^Hb J-Kaohsiung is associated with Hb E.

Among the 19 different mutations, 6 types of α-globin variants were observed. The most common α-globin variants were Hb Queens (38.46%, 5/13) and Hb Q-Thailand (23.08%, 3/13). The other mutations in order of frequency were Hb Daneshgah-Tehran (15.38%, 2/13), Hb Galliera I (7.69%, 1/13), Hb I (7.69%, 1/13), Hb Thailand (7.69%, 1/13).

Furthermore, eleven types of β-globin variants were observed. The most common β-globin variant found was Hb E (89.78%, 281/313), followed by Hb New York (4.79%, 15/313), Hb J-Bangkok (1.60%, 5/313), Hb G-Copenhagen (0.96%, 3/313), Hb J-Lome (0.64%, 2/313), Hb J-Kaohsiung (0.64%, 2/313), Hb D-Los Angeles (0.32%, 1/313), Hb G-Coushatta (0.32%, 1/313), Hb Hope (0.32%, 1/313), Hb Köln (0.32%, 1/313), and a novel β-globin variant (0.32%, 1/313). We named this novel β-globin variant (HBB:c.148 T > C, p.Ser49Pro) as Hb Yunnan.

Supplementary Tables-globin variant were identified: one case of HBD:c.394 C > G (p.Gln131Glu) and one case of HBD:c.198 G > T (p.Lys65Asn). They are two novel δ-globin variant firstly identified in this study and we named as Hb A_2_-Puer and Hb A_2_-Yunnan, respectively. The haematological and electrophoretic data of these two Hb variants are summarized in Supplementary Table 2.

### Bioinformatics analysis of hemoglobin variants

Conservation analysis of β-globin sequences (Supplementary Fig. 4), showed that the Val residue at CD98 (Val98) was evolutionarily highly conserved among the sequences analyzed in our study when compared across multiple species. In our analysis, four amino acid residues were relatively evolutionarily conserved and the remaining five amino acid residues were variable. For α-globin, three amino acid residues were evolutionarily conserved, two amino acid residues were average, and one amino acid residue was variable. In addition, the predicted pI of all 19 Hb variants are summarized in Table 1.

The pathogenicity prediction of all 19 variants obtained by SIFT and Polyphen-2 prediction models showed a discrepancy in our results as seen in Table 2. Here, 15 variants were found to be “deleterious” to protein function and the remaining four variants were characterized as being “benign” by SIFT analysis. In contrast, eight variants were predicted to be “benign” by PolyPhen-2, four variants were found to “possibly damaging”, and seven variants were predicted to be “damaging”.

**Figure 1:**
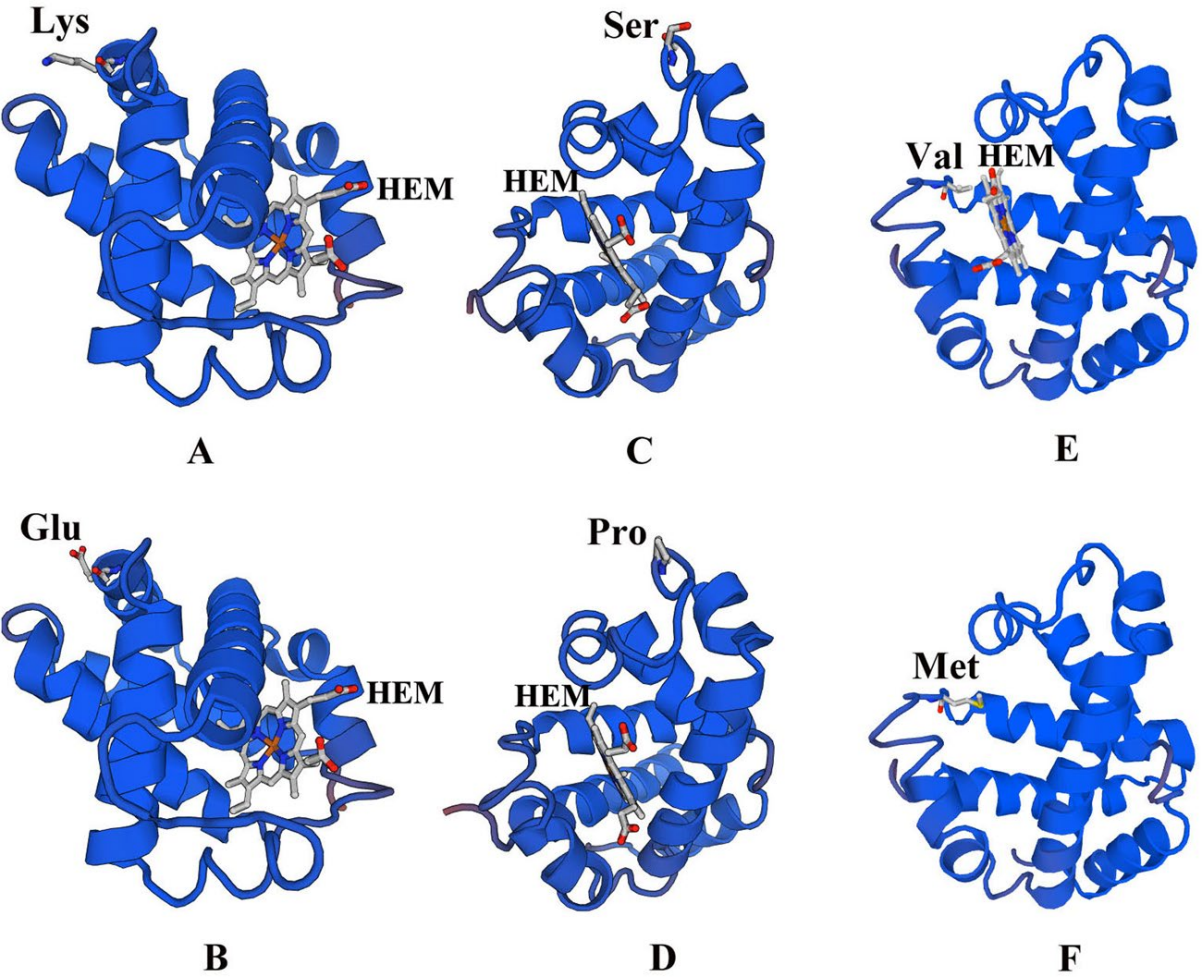
Three-dimensional structure of tetramer Hb A simulated by SWISS-MODEL prediction. Both the Lys16 (**A**) and Glu16 (**B**) were predicted to form α-helix. Both the Ser49 (**C**) and Pro49 (**D**) were predicted to form coil. Models for Met98 (**F**) can not contain HEM and are not the same one as Val98 (**E**).

Supplementary Tables. Based on the typicality and novelty of the Hb variants found, we selected Hb I, Hb Köln, and Hb Yunnan for further analysis. The haematological and electrophoretic characterization of the three patients selected are demonstrated in Supplementary Table 2. A comparison of the globin subunit active sites of the native and the mutant proteins for the three mutations are summarized in Fig. 1. Figure 1A,B show how the Ser49 and Pro49 residues are predicted to form a coil, respectively. Our analysis suggest that the Ser49Pro mutant does not have a significantly altered tertiary structure when compared to the wild-type Hb A protein. Similarly, for the Hb I variant, both the Lys at CD16 (Lys16) and the Glu at CD16 (Glu16) residues were predicted to be located on the α-helix structure according to the SWISS-MODEL and RaptorX software analysis, which indicated that no changes in the tertiary protein structure of Hb I were observed after the Lys residue replacement by Glu (Fig. 1C,D). On the other hand, the position of the Val98 residue was located on the ligand in contact with the protoporphyrin IX complex containing ferrous iron, which then becomes a heme (HEM) molecule (18 residues in the β-globin chain were described as DNA-binding sites for HEM), according to our SWISS-MODEL analysis. As shown in Fig. 1F, the 3D models for the Met98 residue evaluated by the SWISS-MODEL cannot contain HEM molecules, indicating a loss of the oxygen-carrying capacity for the Hb Köln mutant.

**Figure 2:**
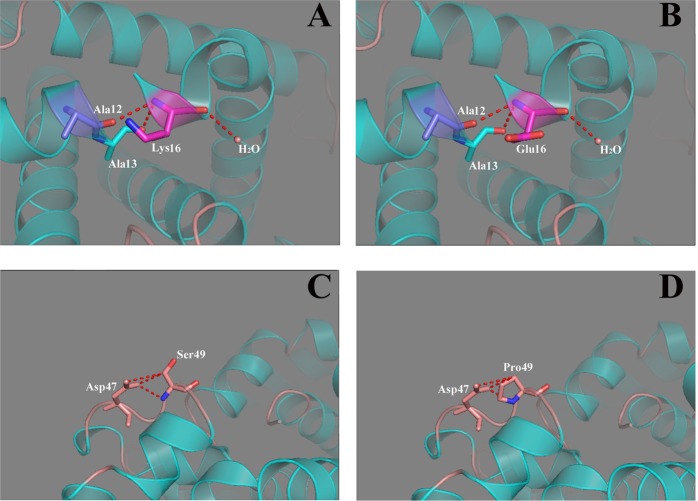
Close-up view of mutation sites and its local environment caused by the change of amino acid. (**A**) Normal Lys16; (**B**) mutated Glu16, both Lys16 and Glu16 connected with the aromatic side chains of Ala12 and Ala13. (**C**) Normal Ser49; (**D**) mutated Pro49, both Ser49 and Pro49 connected with the aromatic side chains of Asp47.

In order to provide further support for these results, the 3D structures for the Hb I and the Hb Yunnan variants were further assessed by the PyMOL software. As seen in Fig. 2A, the Lys16 residue is located on the α-helix, with its side chain connected to the aromatic side chains of the Ala12 and Ala13 residues. And a mutation from Lys to Glu was found to maintain this structure. The structure of the mutant tetramer was almost identical to those of the native Hb molecule as observed by PyMOL analysis (Fig. 2B). We have also modelled the novel Ser49Pro mutant Hb molecule, which was found engaged in a similar pattern of interaction as with the wild-type molecule (Fig. 2C,D).

## Discussion

As seen in this study, a combined analysis of CE and DNA sequencing can help accurately diagnose Hb variants and better provide treatment options for associated haemoglobinopathies. Genetic diversity mapping, complete genotype-phenotype associations, and bioinformatic analyses of Hb variants, have been shown to be useful in guiding haemoglobinopathy control and treatment options in the population of Southern China. In our study, 41,933 subjects from 16 prefecture-level divisions throughout the Yunnan province attended the screening programs for Hb variants. Among the 41,933 subjects investigated, 19 types of Hb variants have been found in 328 cases. The prevalence (0.78%) was higher than the frequency of variants found in the Guangdong province (0.358%), as previous reported^10^, but lower than the frequency found in the Yunnan population that was reported thirty years ago^7^. The discrepancies observed in the frequency of Hb variants from these two studies of the Yunnan province population might have resulted from two possible phenomena: (1) more accurate and rational application strategies based on a larger population sample analysis was used to screen Hb variants in our study; (2) a more diverse population due to heavy migration over the last years (more than 20% of the population has now originated in other provinces, see Supplementary Fig. 1), can result in a change of variant frequency and compound heterozygosity, similar result also be reported previously^8^.

Importantly, 483 subjects (1.15%, 483/41,933) were screened positively by CE, and 328 cases (0.78%, 328/41,933) were confirmed to be Hb variant carriers. For the undiagnosed 155 cases with positive samples of abnormal Hb bands, we predict the following: (1) some samples might have resulted positive from other globin mutations such as γ-globin^18^; and (2) some small confounding Hb bands might have been the result of sample degradation or contamination. On the other hand, CE methods can not fully detect all Hb variants. In our another unpublished research, we found that Hb-Hamilton variants can not be characterized independently and seem to co-migrate with the Hb A variant band using the CE system.

Furthermore, it can be predicted that these Hb variants are the mutated Hb A variants with different electrophoresis velocity, depending on their pI, as well as the molecular weight and structure of the protein. The predicted pI of each Hb variant was calculated by website software (Table 1). The separate profiles of the Hb variants obtained with CE methods were summarized in Table 1 for further molecular testing. Some Hb variants were clearly separated at one zone. In contrast, other Hb variants were displayed in two zones. And some other variants cannot be completely differentiated from the Hb A variant, such as Hb Hope, Hb Galliera I, and Hb Yunnan (Supplementary Fig. 3).

In this study, six types of α-globin, 13 types of β-globin, and two types of δ-globin variants were observed. The most common α-globin variants observed were the Hb Queens (38.46%) and the Hb Q-Thailand (23.08%) variants. In addition, the most common β-globin variants observed were the Hb E variant (89.78%), followed by the Hb New York variant. This spectrum is very similar to the results obtained in Southern China and other analysis of nearby countries^10,19,20^. In addition, there rarely has been an Hb variant resulting from δ- or γ-globin mutations^3^, which can be explained by two possibilities: (1) the Hb A_2_ (α2δ2) constitutes only a small proportion of the total Hb content, so it could be easily missed in the plasma^21^. (2) γ-globin is mainly expressed in the fetus during development and in newborns, which is rapidly diminished after birth. Consistently, most Hb variants resulting from γ-globin have been found expressed in newborns, but not in adult subjects^18^. In addition, in this study, we found 48 cases of αα^CS^ and 14 cases of Hb H or Hb Bart’s variants. Usually, αα^CS^, Hb H, and Hb Bart’s have not been considered to be Hb variant^2^.

For the 19 rare mutations observed in this study, the Hb Yunnan, Hb A_2_-Puer, and Hb A_2_-Yunnan variants resulted from the three novel mutations observed here for the first time. So far, the Hb E, HB New York, Hb J-Bangkok, and Hb Queens variants have been the most popular mutations in the Yunnan population. The Hb E, Hb Queens, Hb Q-Thailand, Hb Thailand, Hb J-Bangkok, and Hb J-Lome variants have been mainly prevalent in the Southeastern Asia and Southern China regions^22–27^. The Hb New York variant can be found in Chinese and American subjects^28^, while the Hb J-Kaohsiung variant has been mostly encountered in Thailand and Chinese populatons^29^. Moreover, the Hb Daneshgah-Tehran variant has been reported in an Iranian and an Argentinian family^30^, while the Hb I variant was found in an American and a Chinese family^31^. Similarly, the Hb Galliera I variant, has been found expressed in an Italian family (irretrievable published literature), while the Hb D-Los Angeles variant has been found widespread globally^32^. Notably, the Hb Köln variant was found sporadically distributed around the world as a *de novo* mutation^33^. Additionally, the Hb G-Copenhagen variant was found expressed in European populations^34^, and the Hb G-Coushatta and Hb Hope variants fortuitously prevalent in both European and Asian populations^35,36^.

The pathogenicity effects of Hb variant missense mutations can be predicted preliminarily depending on the conservation of the amino acid sequence, as important amino acids should be conserved across different species^37^. As seen in online Supplementary Fig. 4, the Val98 residue (V98) of the β-globin chain in the Hb Köln variant is a highly conserved, mutation that on this site of the protein should be “deleterious”. In contrast, some amino acids such as Glu at the CD22 residue of the β-globin chain (i.e., E22, Hb G-Coushatta) and Leu at the CD34 residue of the α-globin chain (i.e. L34, Hb Queens) are variable, as mutations on this site of the protein should be “benign”.

There are 15 variants were found to be “deleterious” by SIFT analysis and seven variants were predicted to be “damaging” by the PolyPhen-2 software prediction model. Some Hb variants such as Hb E, Hb J-Bangkok, and Hb New York had no apparent clinical effects reported by previous report^22,26,28^. And individual of δ-globin gene defects usually has no clinically meaningful problems for low concentration of Hb A_2_ regardless of the results of prediction^9,11^. But all of them were predicted to be “damaging” by SIFT and PolyPhen-2. These results indicated that these software models were not completely reliable in their capability to predict the pathogenicity of Hb variant. These results also show that it remains difficult to make accurate predictions from point mutations, based solely on software analysis, as reinforced by a previous report^13^. A better approach is to use a combination of several bioinformatics tools and clinical data analysis to accurately understand the phenotypic effects of these Hb mutations on a particular subject’s molecular profile.

Furthermore, we modelled the structure of the three rare or novel mutations found in this study. The position of the Val98 residue on the α-helix protein structure is indicative of the meaningful impact the HEM interaction has on protein function. As shown in Fig. 1F, the 3D models for the Met98 residue as evaluated by the SWISS-MODEL showed that it cannot contain a HEM complex, indicating that the oxygen carrying function fulfilled by the HEM complex is completely lost in this mutation, presumably leading to obvious anemia for the carrying subject. Therefore, we could reason that the Val98 residue is a pivotal one, affecting the HEM-binding site and making the Hb Köln variant, a “deleterious” one. Similarly, a “deleterious” mutation was observed in the Hb Debrousse variant, where the connection between Leu at the CD96 residue (Leu96) with HEM was also disrupted^38^.

As it can be seen from the results of the RaptorX and SWISS-MODEL analysis, secondary protein structures cannot be disrupted for p.Lys16Glu and p.Ser49Pro. Furthermore, PyMOL software analysis results show that the atom connections of both mutant tetramers were barely modified compared to normal tetramers, indicating a functional retention of proteins. Additionally, no further information supported the fact that these two amino acids (Lys16 and Ser49) were positioned in a complex or were part of an important interaction network. Here, we infer that these two mutations do not give rise to an appreciable functional and structural change in the tetramer Hb A variant, which is further reinforced by the hematological and clinical features observed in the patients carrying the Hb A variant, who were studied in this research.

In conclusion, our study demonstrates an useful way to determine a detailed and accurate prevalence and molecular characterization of Hb variants in the Yunnan province population. Importantly, significant differences were observed in the molecular spectrum and frequency of Hb variants in the Yunnan subjects who enrolled in our study. Characterizing the structure and function of Hb variants is critical in prenatal diagnosis and control programmes for thalassaemia. The application of a combined molecular approach with clinical data and efficient bioinformatics tools as described in this report, will enable a guideline for functional studies and prenatal diagnosis to be developed as basis for future studies.

## Supplementary information

Supplementary Information

## Data Availability

All data generated or analysed during this study are included in this published article (and its Supplementary Information files). Other datasets generated during and/or analysed during the current study are available from the corresponding author on reasonable request.
