# Supplementary material for: Molecular epidemiology, pathogenicity, and structural analysis of haemoglobin variants in the Yunnan province population of Southwestern China
Source: Sci Rep. 2019 Jun 4;9:8264. doi: 10.1038/s41598-019-44793-0 (PMC6547717; doi:10.1038/s41598-019-44793-0)
Supplement: Supplementary file 1 — Supplementary Information [file 41598_2019_44793_MOESM1_ESM.pdf]

Supplementary Information

Manuscript Title

Molecular epidemiology, pathogenicity, and structural analysis of haemoglobin variants in the Yunnan province population of Southwestern China

Authors

Jie Zhang, Peng Li, Yang Yang, Yuanlong Yan, Xiaohong Zeng, Dongmei Li, Hong Chen, Jie Su and Baosheng Zhu*

Detail of submitted information

Figures Supplementary S1 to S4

Tables Supplementary Table S1 to Table S2

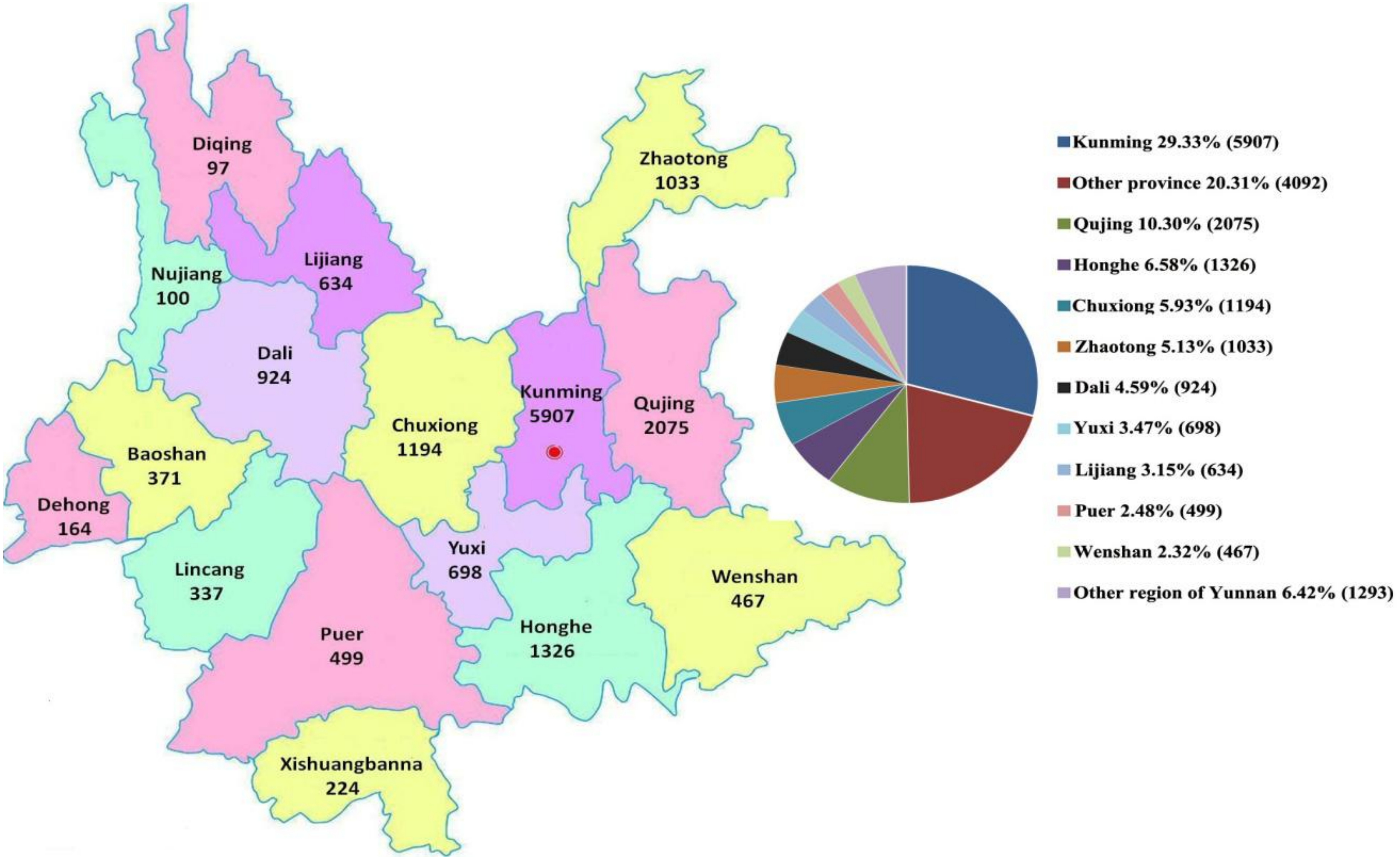

Supplementary Figure S1 List of the 20,142 specimens registered according to their region of origin.

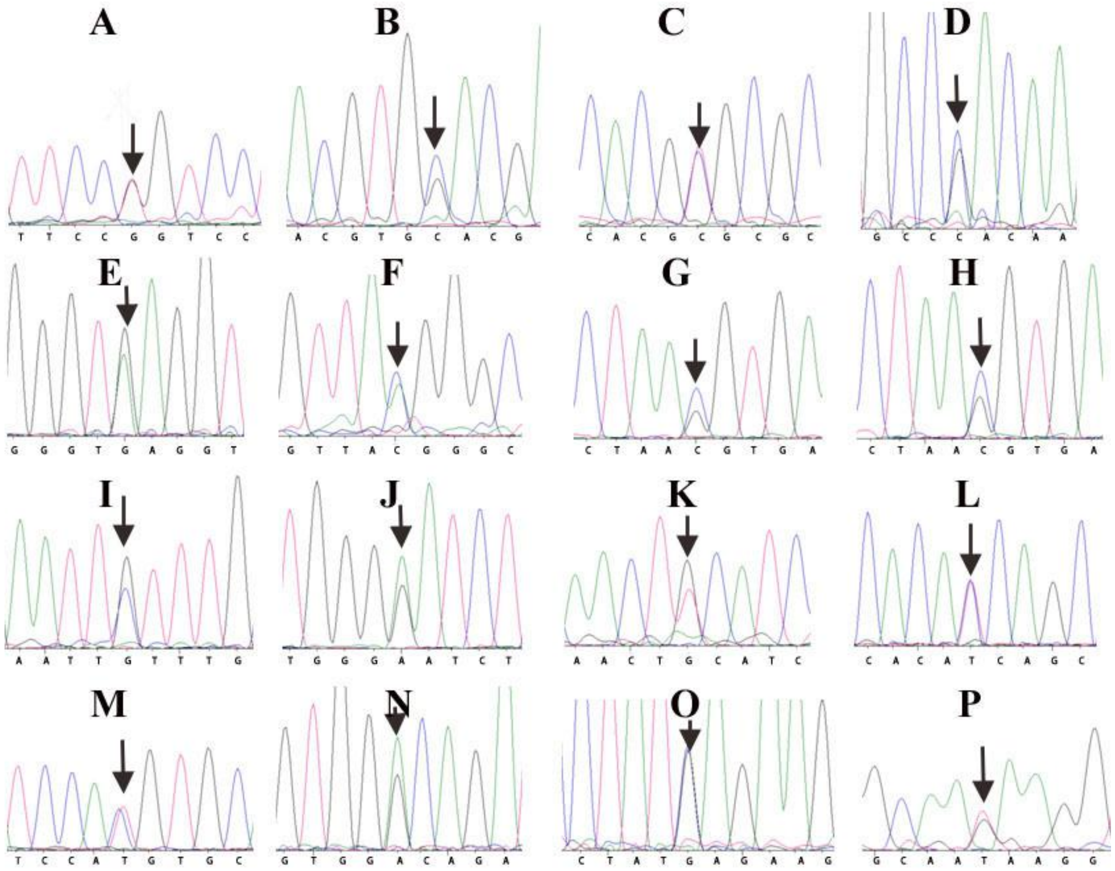

Supplementary Figure S2 DNA sequencing profiles of the Hb variants analyzed. Sequencing profile of (A) Hb Queens, (B) Hb Q-Thailand, (C) Hb Daneshgah-Tehran, reverse sequencing, (D) Hb Galliera I, (E) Hb I, (F) Hb Thailand, (G) Hb J-Lome, (H) Hb J-Kaohsiung, (I) Hb D-Los Angeles, reverse sequencing, (J) Hb G-Copenhagen, (K) Hb G-Coushatta, reverse sequencing, (L) Hb Hope, (M) Hb Köln, (N) Hb Yunnan, (O) Hb A_2-Puer, and (P) Hb A_2-Yunnan, as seen accordingly in this 16-panel figure.

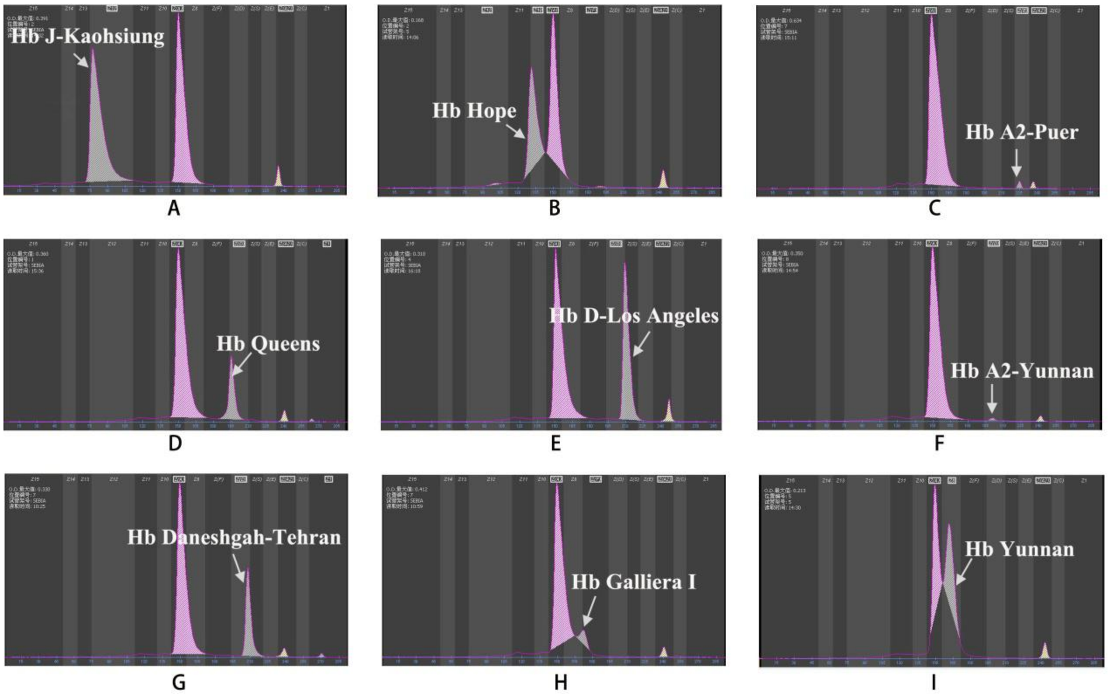

Supplementary Figure S3 Capillary electrophoresis profiles of potentially confounding or novel Hb variants.

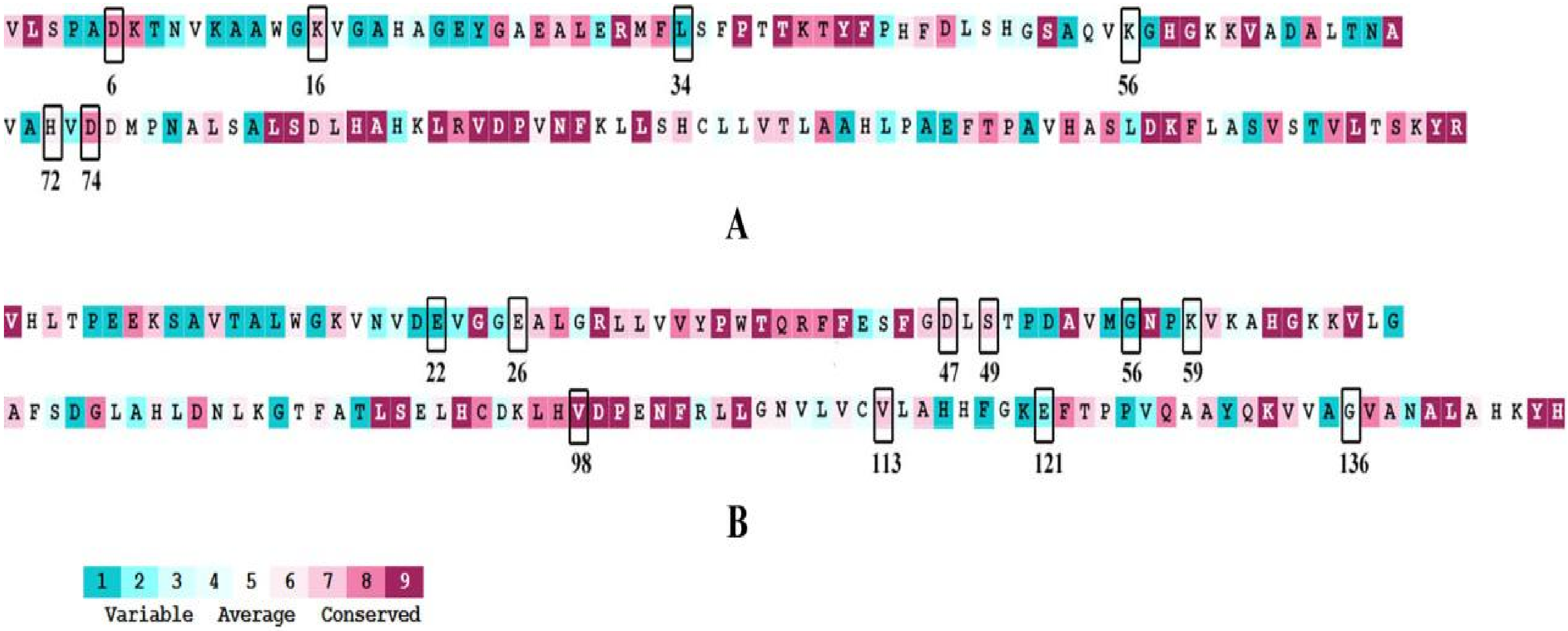

Supplementary Figure S4 Schematic diagram depicting the conservation of Hb variants between species. The degree of conservation is shown in colors ranging from 1 to 9: variable (1-3), average (4-6), or conserved (7-9) degrees. (A) Distribution of amino acids along the HBA protein. The site of L34 is variable across species (ranged as 1), whereas the distribution of amino acids along the HBB protein (B), shows that the V98 site is highly conserved (ranged as 9).

Table S1 Haematological and electrophoretic characterization of several novel and rare Hb variants classified by gender and age

Type	Gender	Age	Hb (g/L)	MCV (fl)	MCH (pg)	Hb A (%)	Hb A_2 (%)	Hb F (%)	Hb X (%)
Hb Yunnan	Female	29	133.0	86.3	28.0	50.4	5.6	-	44.0
Hb I	Female	39	123.0	92.2	29.1	79.8	1.9	-	18.3
Hb Köln	Female	39	73.0	98.3	27.5	91.2	3.7	1.3	3.8
Hb A_2-Puer	Male	35	161.0	85.2	29.0	97.0	1.3	-	1.4
Hb A_2-Yunnan	Female	31	138	91.0	30.3	98.0	1.3	-	0.7

Notes: Hb, haemoglobin; MCV, mean corpuscular volume; MCH, mean corpuscular haemoglobin; femtoliters; pg, picograms.

Table S2 Globin primer sequences and expected product size

Globin	Sequence	Annealing temperature (°C)	Product size (bp)
α1	F1: 5'-TCCCCACAGACTCAGAGAGAACC-3'	66	889
	R1: 5'-CCATGCCTGGCACGTTTGCTGAG-3'		
α2	F2: 5'-TCCCCACAGACTCAGAGAGAACC-3'	66	889
	R2: 5'-AACACCTCCATTGTTGGCACATTCC-3'		
β1	F1: 5'-AGGTACGGCTGTCATCAC-3'	55	746
	R1: 5'-TGCAATCATTCGTCTGTTTC-3'		
β2	F2: 5'-GCCTCTTTGCACCATTCTA-3'	55	659
	R2: 5'-GTTTGCAGCCTCACCTTC-3'		
δ1	F1: 5'-CTGAGTCAAGACACACATGAC-3'	58	947
	R1: 5'-TGGTATGCATAATTTGAGTTGTTG-3'		
δ2	F2: 5'-AATATCCTGTCTTTCTCTCCCAAC-3'	58	1133
	R2: 5'-TAATTTCTGCTCTTTGGAGGTAG-3'		

Notes: F, forward; R, reverse; bp, base pair.
